# Working Towards Gender and Racial Diversity in Pediatric Residency Programs in the United States

**DOI:** 10.7759/cureus.21633

**Published:** 2022-01-26

**Authors:** Sundas Saboor, Sadiq Naveed, Beenish Safdar, Amna M Chaudhary, Sonia Khan, Faisal Khosa

**Affiliations:** 1 Department of Public Health, Harvard T.H. Chan School of Public Health, Boston, USA; 2 Department of Psychiatry, Hartford Hospital, Institute of Living, Hartford, USA; 3 Department of Psychiatry, Elmhurst Hospital Center, Icahn School of Medicine at Mount Sinai, New York, USA; 4 Department of Psychiatry, University Hospitals, Case Western Reserve University, Cleveland, USA; 5 Department of Psychiatry, Frontier Medical & Dental College, Abbottabad, PAK; 6 Department of Radiology, Vancouver General Hospital, Vancouver, CAN

**Keywords:** diversity, residency, racial, gender, pediatrics

## Abstract

Introduction

The gender and racial profile of the pediatric population in the United States (US) is more diverse than that of the pediatricians that cater to their healthcare needs. Gender and racial diversity remains limited among pediatric residents and fellows, faculty, and leadership. Our study objectives were to explore the gender and racial disparity among pediatric residents in the US.

Methods

This was a retrospective analysis of the Accreditation Council for Graduate Medical Education (ACGME) database. The database encompassed all residents in US pediatrics residency programs from 2007 to 2021, categorizing them into White (non-Hispanic), Asian/Pacific Islander, Hispanic, African American/Black (non-Hispanic), Native American/Alaskan, others (races not included in the mentioned categories), and unknown. Gender was grouped into male, female, and not reported.

Results

From 2011 to 2021, the greatest increase in relative change (%) was seen for Asian or Pacific Islander (+58.42%), followed by Black (non-Hispanic) (+45.24%), White (non-Hispanic) (+43.37%), and Hispanic (+42.18%) races. The Native American/Alaskan relatively decreased 50%. The representation of female residents relatively increased by 13.27% as compared to the relative increase of male residents (+14.77%) from 2007 to 2021.

Conclusion

It is imperative to have a healthcare workforce that is representative of the existing communities in the US in terms of race, ethnicity, and gender to provide culturally sensitive care to the diverse patient population of the US.

## Introduction

A diverse physician workforce addresses healthcare disparity while improving patient outcomes and increasing satisfaction among patients. It fosters greater innovation in health care and facilitates the recruitment of minorities into clinical trials for better treatment solutions [1,2]. Diverse populations require personalized approaches to meet their healthcare needs. With an ever-increasing number of minority patients, more minority physicians are needed so that minority patients can choose to see physicians of their race [2]. Literature has shown that minority doctors are more likely to treat minority patients, who often live in underserved areas [1]. Racial concordance, by a diverse physician population, encourages physician understanding of the cultural, social, and economic factors that influence patients and can foster trust and communication [2].

Despite these advantages, the proportion of individuals belonging to racial/ethnic minority groups in health care is not comparable to their representation in the overall United States (US) population. In 2019, the percentage of Black/African American, American Indian or Alaska Native, Native Hawaiian or other Pacific Islander, and Hispanic or Latino was more than about 33%, while these groups comprised approximately 12% of US medical school graduates in the same year [3].

The knowledge and understanding of serving individuals with diverse racial and ethnic backgrounds, different values, health beliefs, and alternative perspectives about health and wellness are crucial. Although healthcare systems have continued to diversify with an increased number of ethnic and racial employees, underrepresented minorities in medicine (URiM) and women continue to face challenges regarding their propionate presentation within the healthcare workforce [4]. Healthcare systems heavily depend on female physicians, still, they discriminate against women and tend to concentrate female physicians to work in lower occupations globally [5,6]. On the other hand, race is a social construct introduced by the society and is not a biological construct that reflects innate differences [7]. Currently, 48% of medical students in the US are women whereas only 35% of the physician workforce is represented by women [8]. Women and URiM also struggle to progress to the leadership and senior faculty positions due to gender stereotypes and unconscious biases that plague women and URiM in medicine [9]. Women and URiM are underrepresented in medical leadership, they are given less funding than their counterparts for research/grants, and they are promoted at lower rates. They are more likely to experience implicit and explicit bias, reduced career flexibility, and isolation and exclusion from opportunities to advance in medical careers [9,10].

The racial, ethnic, and gender disparities in health care are known to limit access to care and the earlier onset of illness, more severe disease, and poor quality of care that arise from differing socioeconomic conditions (measured by income, education, or occupational status) as compared to their peers. Evidence suggests that even after such disparities are accounted for, race and ethnicity remain significant predictors of the quality of health care received [11]. Recent data suggest that although 38.7% of the US population consists of racial and ethnic minorities, African Americans, Hispanics/Latino, and Native Americans make up only 4%, 4%, and <0.04% of medical doctors, respectively [12].

According to the American Medical Association (AMA), the number of female pediatric residents has gradually increased (+75%), but ethnic minority races recruitment among all specialties especially pediatrics continues to be low as compared to their representation in the general population [13].

Many studies have been done in recent years to analyze the recruitment of URiM in pediatric residency programs [14,15]. However, in this study, we explored the trends of recruitment of residents not only according to race but also the percentage of women in the US pediatric residency programs from 2007-2008 to 2020-2021. The primary objective of this study is to help the policymakers understand the longitudinal trends, thereby enabling them to address these disparities through evidence-based strategy.

## Materials and methods

We retrospectively analyzed the data extracted from the annual Accreditation Council for Graduate Medical Education (ACGME) Data Resource Books from 2007 to 2021 [16].

Variables

For pediatric residency training programs, demographic data (i.e., race/ethnicity and gender) of residents were extracted. Race/ethnicity was categorized as self-reported White/non-Hispanic, Asian/Pacific Islander, Hispanic, Black/non-Hispanic, Native American/Alaskan, others, and unknown. The Asian category was separated from the Pacific Islander category in the 2018-2019 ACGME Resource Book. Asian and Hawaiian Native/Pacific Islander have a separate category from 2019-2021 ACGME Data Resource Book. However, for the purpose of analysis, they have been combined in our research. According to the ACGME Data Resource Books, "Unknown" ethnicity includes both unknown and blank, while "Other" indicates a known ethnicity other than the ones available for selection [16]. Gender was grouped into male, female, and not reported.

Data analysis

We analyzed the data by gender and racial distributions and its temporal trend by year and across the specialty of pediatrics. Absolute and relative percentage changes were calculated to highlight trends in resident appointments over time and across specialties; absolute change is the actual difference in the total number of residents from 2007 to 2021 (gender) and 2010 to 2021 (race) and relative change is the change (%) in pediatric residents relative to 2007 in case of gender and 2010 in case of race. Although data for gender distribution were available for all years (i.e., 2007 to 2021), race/ethnicity was reported starting from the year 2011.

## Results

Distribution of race/ethnicity in pediatric residents and its temporal trends

From 2011 to 2021, the racial/ethnic trends in US pediatric residents showed an increase for White (non-Hispanic), Asian or Pacific Islander, Black (non-Hispanic), Hispanic race, and others (Table 1). The greatest increase in relative change (%) was seen for Asian/Pacific Islander (+58.42%), Black (non-Hispanic) (+45.24%), followed by White (non-Hispanic) (+43.37%) and Hispanic (+42.18%) pediatric residents. However, Native American/Alaskan pediatric residents relatively decreased by 50% simultaneously.

**Table 1:** Percentage changes in pediatric residents from the year 2011-2012 to 2020-2021 by race according to the Accreditation Council for Graduate Medical Education (ACGME) Data Resource Books. The data are self-reported. "+" denotes increase and "−" denotes decrease.

Race/ethnicity	2011-2012, N (%)	2020-2021, N (%)	Absolute change (%)	Relative change (%)
White, non-Hispanic	3220 (38.06)	4780 (54.57)	+16.51	+43.37
Asian or Pacific Islander	1225 (14.48)	2010 (22.94)	+8.46	+58.42
Hispanic	433 (5.12)	638 (7.28)	+2.16	+42.18
Black, non-Hispanic	400 (4.73)	602 (6.87)	+2.14	+45.24
Native American/Alaskan	20 (0.24)	11 (0.12)	−0.12	−50.00
Others	328 (3.88)	196 (2.23)	−1.65	−42.52
Unknown	2834 (33.50)	522 (5.95)	−27.55	−82.23
Total	8460 (100%)	8759 (100%)		

Gender comparison of pediatric residents and its temporal trends

The representation of women increased in pediatric residency from 2007 to 2021. There was an absolute increase of 8.32% and a relative increase of 13.27% for women pediatric residents from 2007 to 2021 (Table 2). A simultaneous increase was also observed for male pediatric residents for both absolute and relative change (%) by +3.47% and +14.77%, respectively.

**Table 2:** Percentage changes in pediatric residents from the year 2007-2008 to 2020-2021 by gender according to the Accreditation Council for Graduate Medical Education (ACGME) Data Resource Books. The data are self-reported. "+" denotes increase and "−" denotes decrease.

Gender	2007-2008, N (%)	2020-2021, N (%)	Absolute change (%)	Relative change (%)
Male	1913 (23.49)	2540 (26.96)	+3.47	+14.77
Female	5106 (62.69)	6698 (71.1)	+8.32	+13.27
Not reported	1126 (13.82)	181 (1.92)	−11.87	−85.89

## Discussion

This study suggests a relative increase for female pediatric residents by 13.27% compared to a relative increase for male pediatric residents by 14.77% from 2007 to 2021. For racial composition, the highest relative increase for pediatric residents has been observed for the Asian/Pacific Islander (58.42%) followed by Black (non-Hispanic) (45.24%), White (non-Hispanic) (43.37%), and Hispanic (42.18%) from 2011 to 2020. However, Native Americans/Alaskans relatively decreased 50% during the same time.

For women, there has been a substantial increase across various clinical disciplines in the last three decades (1990-2019), including pediatrics [10]. The factors for higher numbers of female pediatric residents are flexibility with job selection, geographic location, autonomy over working hours, more scholarly and educational endeavors, and part-time opportunities [17]. About 63% of pediatricians in the US are women [18]. While it is promising, there is still a considerable lack of equity in professional opportunities. Even in this female-dominated profession, in which they may have greater opportunity to organize and demand equal pay for equal work, women receive 76 cents on the dollar compared to their male counterparts [17].

Our study indicates an increase in Asian/Pacific Islander, Black (non-Hispanic), and Hispanic representation while a decrease in Native American/Alaskan representation in the last decade. This trend is consistent with existing studies [14,15]. Not all Asian subgroups are underrepresented in medicine. East and South Asians make up a higher proportion of physicians and are considered “over-represented.” However, Filipinos, Vietnamese, Cambodians, and others are underrepresented in medicine [19]. The rise in Asian representation can be attributed to substantial gains in the admission rates of minorities in medical school admissions [20]. In 1980, the percentage of Asian, Black, and Hispanic medical school matriculants was 4%, 6%, and 4.9%, respectively. The percentage of Asian, Black, and Hispanic medical school matriculants increased to 21.3%, 7.1%, and 6.3% in 2016, respectively. However, Native American/Alaskan medical school matriculants were 0.4% in 1980, which decreased to 0.3% in 2016 [20]. The reasons for not choosing a career in pediatrics by URiM include decreased exposure to academic opportunities in early careers, higher financial burdens, educational debt, experiences of bias and harassment, poor recruitment efforts, and a smaller number of racially/ethnically concordant peers and mentors [21].

The US needs a multifaceted collaborative approach to eliminate healthcare disparities and increase diversity in the healthcare workforce [22]. It is imperative to recruit and retain diverse employees for successful healthcare systems that better understand, reflect, and meet the healthcare needs of the diverse communities [4]. Many medical institutes have taken initiatives to increase diversity among medical school matriculants by allocating financial resources and establishing diversity councils [23]. Although these steps temporarily assisted in improving diversity among residency programs; however, these are not sufficient to achieve diversity, equity, and inclusion in residency training programs. More research is needed that can help devise interventions to enhance the experience of URiM and reduce barriers in academia [24].

The organizations need to add minority faculty in the resident selection committees to help the program directors prescreen the URiM applicants. When possible, URiM faculty members should interview URiM medical students [24]. The residency programs need to revise the scoring rubric to deemphasize United States Medical Licensing Examination (USMLE) scores, grades, Medical College Admission Test (MCAT), and Alpha Omega Alpha (AOA) memberships and develop standardized interview questions. Diversity-led committees should be consulted to review URiM applicant pool for interviews. To minimize bias based on visual appearances, photographs should be discouraged and names should be omitted from resumes during review and ranking processes. Training in diversity for unconscious bias by a professional trainer should be provided in-person and remote [25]. Such initiatives can help to increase diversity among all medical residency programs, including pediatrics.

Limitations

There are limitations to our study. In the context of gender disparity, the non-binary option was not included in the ACGME Data Resource Books until 2020-2021. There is also a limitation of gender fluidity or non-binary/binary options data. Also, we analyzed the data from the annual ACGME Data Resource Books, which relies on participants’ willingness to share their personal information. Since race is a complex social category with evolving meaning, the participants can select more than one race/ethnicity, potentially overestimating the percentage of residents who identify as URiM. There was no information about the combined gender and race of the pediatric residents, e.g., White men/women and Black (non-Hispanic) men/women. There was no detailed information about the races in the unknown and others category.

## Conclusions

The representation of female pediatric residents relatively increased as compared to male pediatric residents from 2007 to 2021. From 2011 to 2021, Asian or Pacific Islanders increased, followed by Black (non-Hispanic), White (non-Hispanic), and Hispanic races; however, the Native American/Alaskan relatively decreased. To improve diversity within the pediatrician workforce, we need to enforce effective recruitment initiatives and provide organizational support systems to support women and URiM during pediatric residency training. We need to create a culturally sensitive healthcare workforce that values diversity and inclusivity and enhances productivity.
